# Acute endocarditis in a pregnant patient requiring post-partum emergency mitral valve repair: a case report

**DOI:** 10.1093/jscr/rjad441

**Published:** 2023-08-08

**Authors:** Ramanish Ravishankar, Azar Hussain, Mahmoud Loubani, Mubarak Chaudhry

**Affiliations:** Faculty of Public Health and Policy, London School of Hygiene and Tropical Medicine, London, UK; Department of Cardiothoracic Surgery, Castle Hill Hospital, Hull, UK; Department of Cardiothoracic Surgery, Castle Hill Hospital, Hull, UK; Department of Cardiothoracic Surgery, Castle Hill Hospital, Hull, UK

**Keywords:** Infective Endocarditis, Mitral Valve repair, Pregnancy, cardiac surgery

## Abstract

A 37-year-old pregnant patient presented with symptoms of shortness of breath, cough and malaise at 36 weeks’ gestation. Antibiotics were started because of suspected bilateral pneumonia. A lower segment caesarean section was undertaken and significant desaturation lead to intubation of the patient. A CTPA confirmed bilateral pneumonia but also elements of heart failure with a 32 mm dilated pulmonary artery. Severe mitral regurgitation was confirmed with trans-thoracic and trans-oesophageal echocardiogram on Day 5 and emergency mitral valve repair was undertaken for possible infective endocarditis (IE) as per the modified Duke criteria, which was confirmed intra-operatively. The patient completed 4 weeks of antibiotics and suffered mild memory impairment post-operatively. She was discharged from complex rehabilitation after 6 weeks of hospital stay at her baseline state. This case presents IE in a pregnant patient with no significant risk factors with successful recovery because of prompt diagnosis and management.

## INTRODUCTION

Infective endocarditis (IE) during pregnancy is rare and has a high mortality rate for both the mother and the foetus [1]. This report presents a case of a pregnant patient at 36 weeks’ gestation with acute IE requiring emergency mitral valve debridement and repair.

## CASE REPORT

A 37-year-old pregnant lady was initially admitted to the Women and Children’s hospital on 36 weeks of gestation with symptoms of shortness of breath, cough and malaise. During admission, she was noted to have significant desaturation refractory to high-flow oxygen. Because of bilateral chest crepitations, raised inflammatory markers and prolonged fever, she was commenced on antibiotics with a suspicion of bilateral pneumonia. She underwent an urgent lower segment caesarean section (LSCS) under general anaesthesia because of not tolerating a spinal anaesthetic. However, because of her saturations dropping to 65%, she was intubated. Following delivery, the patient failed to be extubated and required prolonged ventilation in the intensive care unit (ICU).

**Figure 1:**
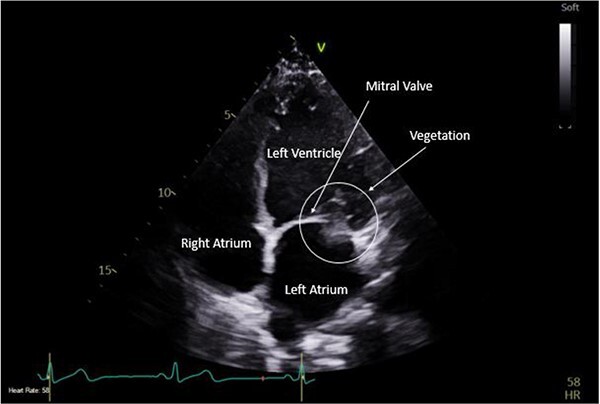
Pre-operative TTE depicting mitral valve with vegetation (labelled).

**Figure 2:**
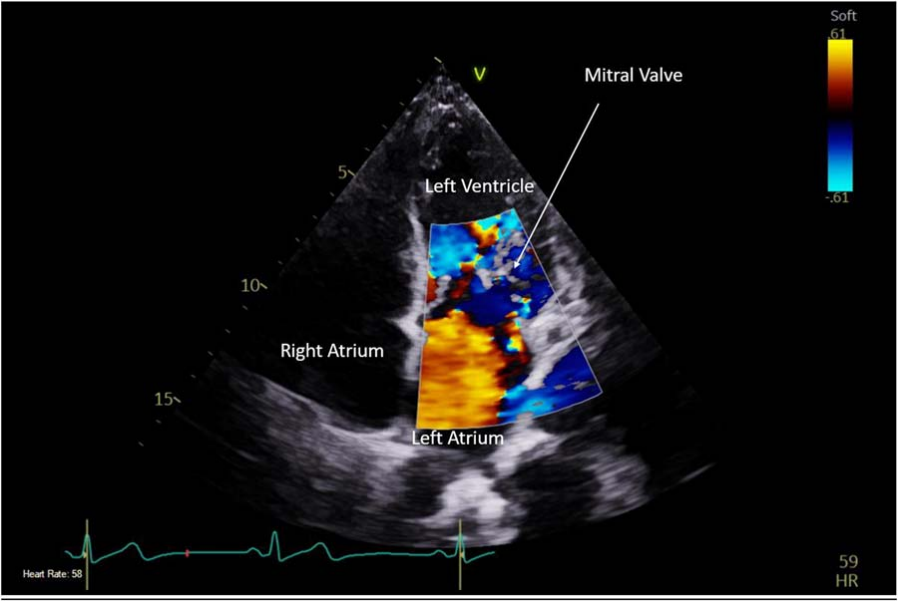
Pre-operative TTE with Doppler depicting mitral valve with vegetation (labelled).

A CTPA was undertaken on Day 2, which excluded pulmonary embolism but confirmed bilateral peri-hilar consolidation and pleural effusions consistent with pneumonia. However, her pulmonary artery was dilated at 32 mm indicative of potential heart failure. On Day 5 of her ICU stay, a transthoracic echocardiogram was undertaken that revealed there to be severe mitral regurgitation including a mobile echogenic mass attached to the underside of the anterior mitral valve leaflet consistent with a vegetation (Figs 1 and 2).

A further trans-oesophageal echocardiogram was undertaken, which confirmed this finding. Coupled with her history of a temperature spike of >38°C, a diagnosis of possible acute IE was made as per the modified Duke criteria, and she was booked for emergency mitral valve repair. She was placed on ceftriaxone, linezolid and erythromycin, and was given spironolactone and furosemide for diuresis.

**Figure 3:**
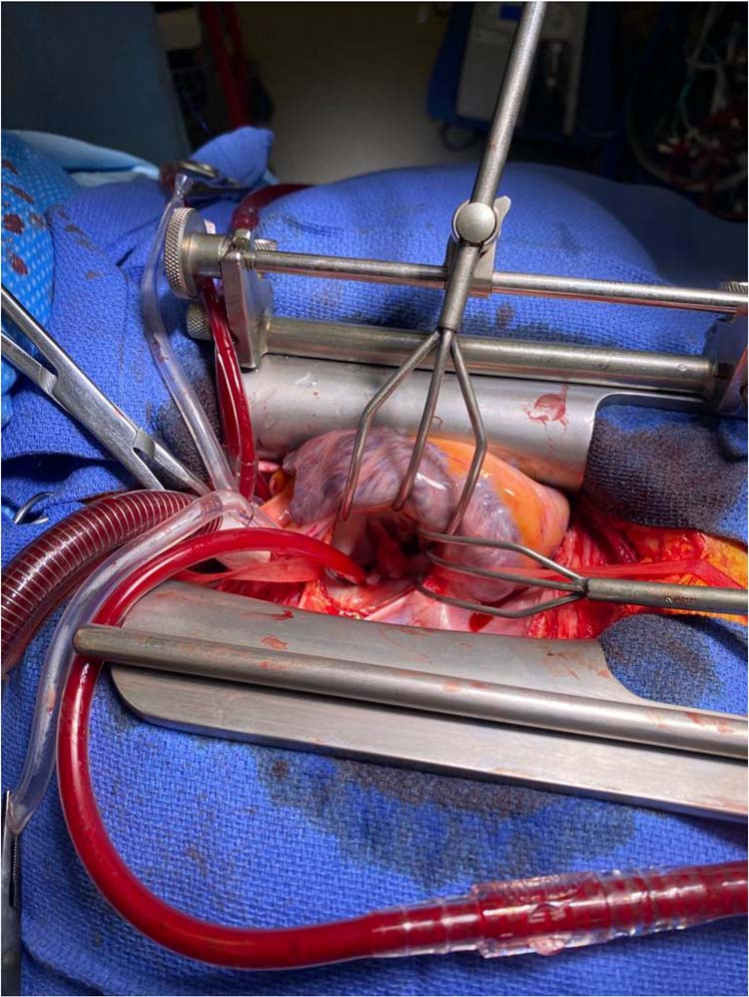
Retracted view of mitral valve with vegetation.

**Figure 4:**
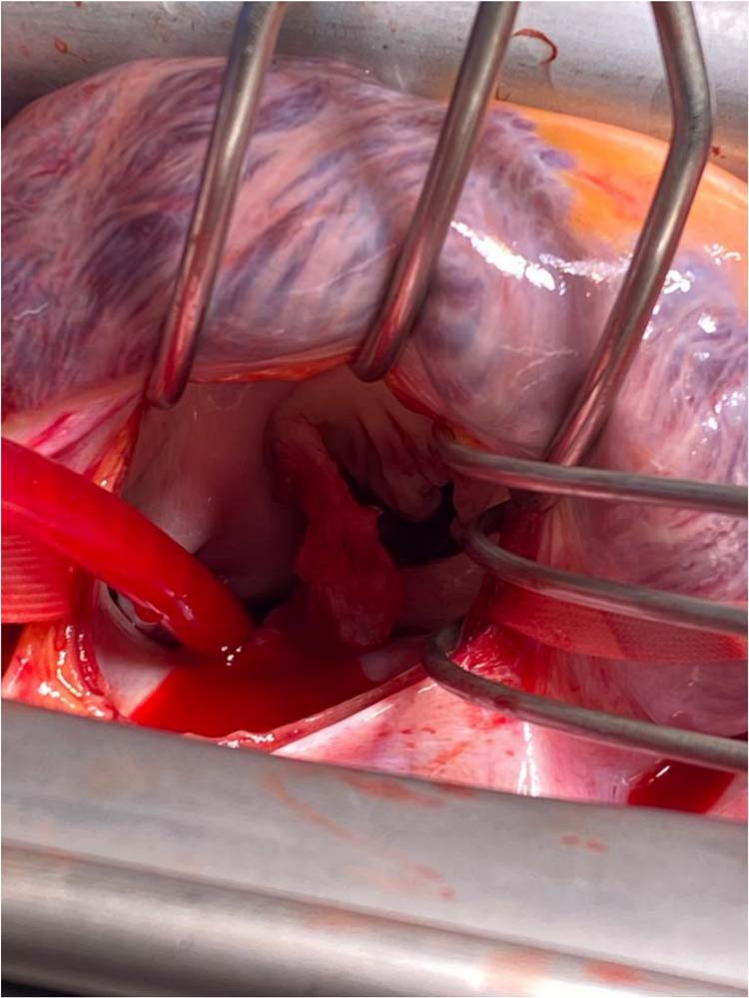
Close-up view of mitral valve with vegetation.

**Figure 5:**
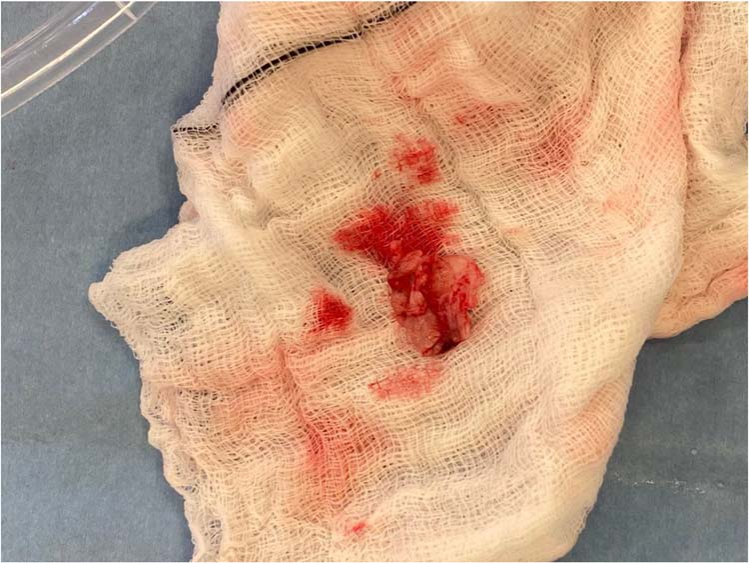
Excised vegetation on a white swab.

**Figure 6:**
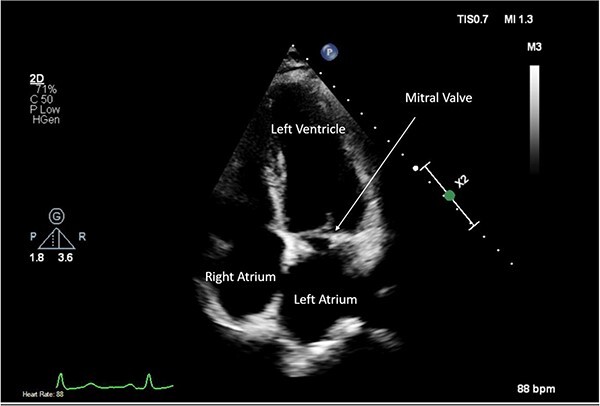
Post-operative TTE depicting mitral valve repair.

**Figure 7:**
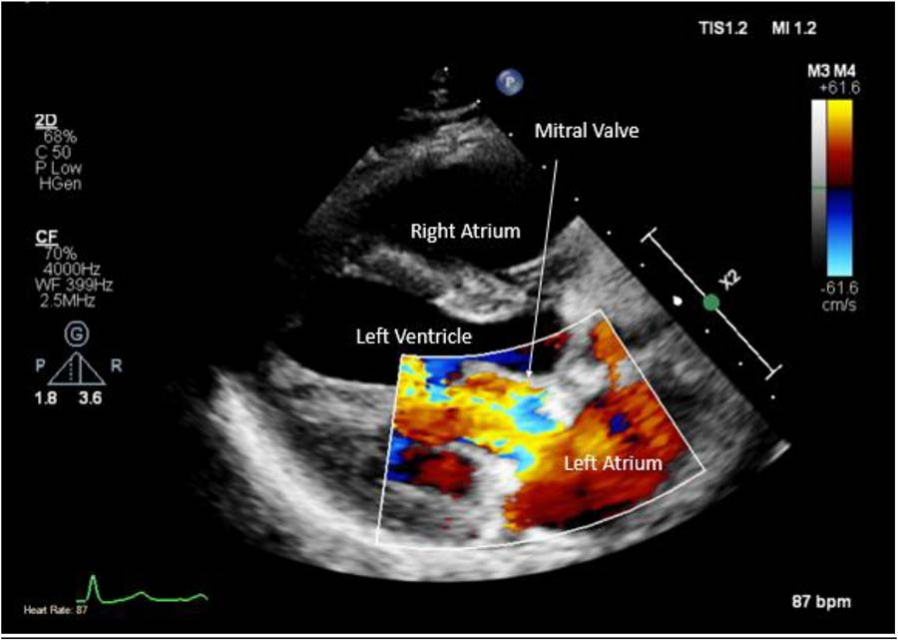
Post-operative TTE with Doppler depicting mitral valve repair.

Intra-operatively, femoral–femoral bypass was established and a 3 cm windsock vegetation was found with a 1.5 × 1.5 cm hole at the aortic inlet on the annular junction. The vegetation travelled from the LV to LA as shown in Figs 3 and 4. The vegetation was excised and the valve debrided, which resulted in moderate regurgitation from severe. Hence, a further 26 mm Physio II ring was placed resulting in no mitral regurgitation. The excised vegetation is shown in Fig. 5 and the post-op TTE images are shown in Figs 6 and 7.

There were no intra-operative complications, and she was commenced on 4 weeks of antibiotic therapy post-operatively. She was noted to have some mild confusion post-operatively and an MRI head was undertaken that confirmed a lacunar infarct. The aetiology of this was attributed to her period of hypoxia during her LCSC or her mitral valve repair. She was transferred to the complex rehabilitation unit and subsequently discharged 6 weeks post-op as she had recovered to her baseline state. She was followed up in clinic 3 months post-discharge and has experienced no complications with excellent recovery.

## DISCUSSION

This is a unique presentation of pregnancy associated IE. The incidence of perinatal IE is incredibly rare; Montoya *et al*. report the incidence of IE in pregnancy to be 0.006% [2]. IE within pregnant women can be even more dangerous leading to consequences such as heart failure and embolization events with a mortality of 33% [1]. One systematic review noted septic pulmonary emboli and CNS emboli to be common complications in perinatal IE [3] with 23.3 and 12.2%, respectively. With such a high morbidity and mortality rate, it is important to utilize a multi-specialty team-based approach in the management of such patients. The European Society of Cardiology (ESC) recommends pregnant patients who present with heart failure because of acute regurgitation to have urgent cardiac surgery because of the complications associated with it.

Previous case reports detailing peri-natal IE have suggested a mixture of early delivery coupled with close maternal monitoring, compared with operative intervention with patients in their first trimester [3]. The adverse effects of cardiopulmonary bypass on pregnant women include premature uterine contraction, increased incidence cerebral and peripheral embolization and an increased association with feto-neonatal death amongst others [4]. Hence, clinical decision-making should be aimed at delivery, and achieving haemodynamic stability post-LSCS thus leading to improved outcomes with cardiac surgery. In this case, the patient was at 36 weeks’ gestation on presentation, and her diagnosis of IE was not made until 5 days post-delivery because of her presentation being masked by a pulmonary infective focus.

Any pregnant patient who presents with a fever and cardiac murmur should have meticulous investigation and IE should be considered as a differential. The ESC Task Force for IE attributes the conception to either a pre-existing cardiac lesion or IVDU. In this patient, she did not have any of the above risk factors and the presence of a concomitant pneumonia masked the underlying issue. However, pregnancy itself can be a contributing factor to IE because of the state of partial immunomodulation [5]. Recent findings have determined that pregnancy modulates the immune response to a pathogen differentially depending on the stages of pregnancy; the first and final stages are described to be pro-inflammatory due implantation, placentation and parturition inducing a strong inflammatory response [6]. Bacterial infection in the third trimester is noted to be more common because of increased cortisol, and neutrophils because of bone marrow demand thus down-regulating the T-cell response. This risk is elevated in the first month post-partum because of the correcting heightened immune response providing an altered and mal-adapted response to infection [7].

In conclusion, this case highlights the importance of assessing for structural heart abnormalities and IE through an echocardiogram in pregnant patients, particularly if presenting with fever and elements of heart failure such as pleural effusion and pulmonary artery dilation. This should also be considered even in the absence of risk factors because of the immunomodulatory effects of pregnancy despite the rare incidence of IE. Prompt investigation and urgent surgical intervention can lead to positive outcomes in these patients.

## Data Availability

The authors confirm that the data supporting the findings of this study are available within the article and its supplementary materials.
